# Lactate-mediated metabolic reprogramming of tumor-associated macrophages: implications for tumor progression and therapeutic potential

**DOI:** 10.3389/fimmu.2025.1573039

**Published:** 2025-05-13

**Authors:** Xiaohan Jin, Ni Zhang, Tinghao Yan, Jingyang Wei, Lingli Hao, Changgang Sun, Haibo Zhao, Shulong Jiang

**Affiliations:** ^1^ Center for Post-Doctoral Studies, Shandong University of Traditional Chinese Medicine, Jinan, China; ^2^ Clinical Medical Laboratory Center, Jining No.1 People’s Hospital, Jining, China; ^3^ Jining No.1 People’s Hospital, Shandong First Medical University, Jining, China; ^4^ Cheeloo College of Medicine, Shandong University, Jinan, China; ^5^ Second College of Clinical Medicine, Shandong University of Traditional Chinese Medicine, Jinan, China; ^6^ College of First Clinical Medicine, Shandong University of Traditional Chinese Medicine, Jinan, China

**Keywords:** tumor microenvironment, lactate metabolism, tumor-associated macrophages, immune regulation, cancer therapy, metabolic reprogramming

## Abstract

The tumor microenvironment (TME) is characterized by distinct metabolic adaptations that not only drive tumor progression but also profoundly influence immune responses. Among these adaptations, lactate, a key metabolic byproduct of aerobic glycolysis, accumulates in the TME and plays a pivotal role in regulating cellular metabolism and immune cell function. Tumor-associated macrophages (TAMs), known for their remarkable functional plasticity, serve as critical regulators of the immune microenvironment and tumor progression. Lactate modulates TAM polarization by influencing the M1/M2 phenotypic balance through diverse signaling pathways, while simultaneously driving metabolic reprogramming. Furthermore, lactate-mediated histone and protein lactylation reshapes TAM gene expression, reinforcing their immunosuppressive properties. From a therapeutic perspective, targeting lactate metabolism has shown promise in reprogramming TAMs and enhancing anti-tumor immunity. Combining these metabolic interventions with immunotherapies may further augment treatment efficacy. This review underscores the crucial role of lactate in TAM regulation and tumor progression, highlighting its potential as a promising therapeutic target in cancer treatment.

## 1 Introduction

**Figure 1:**
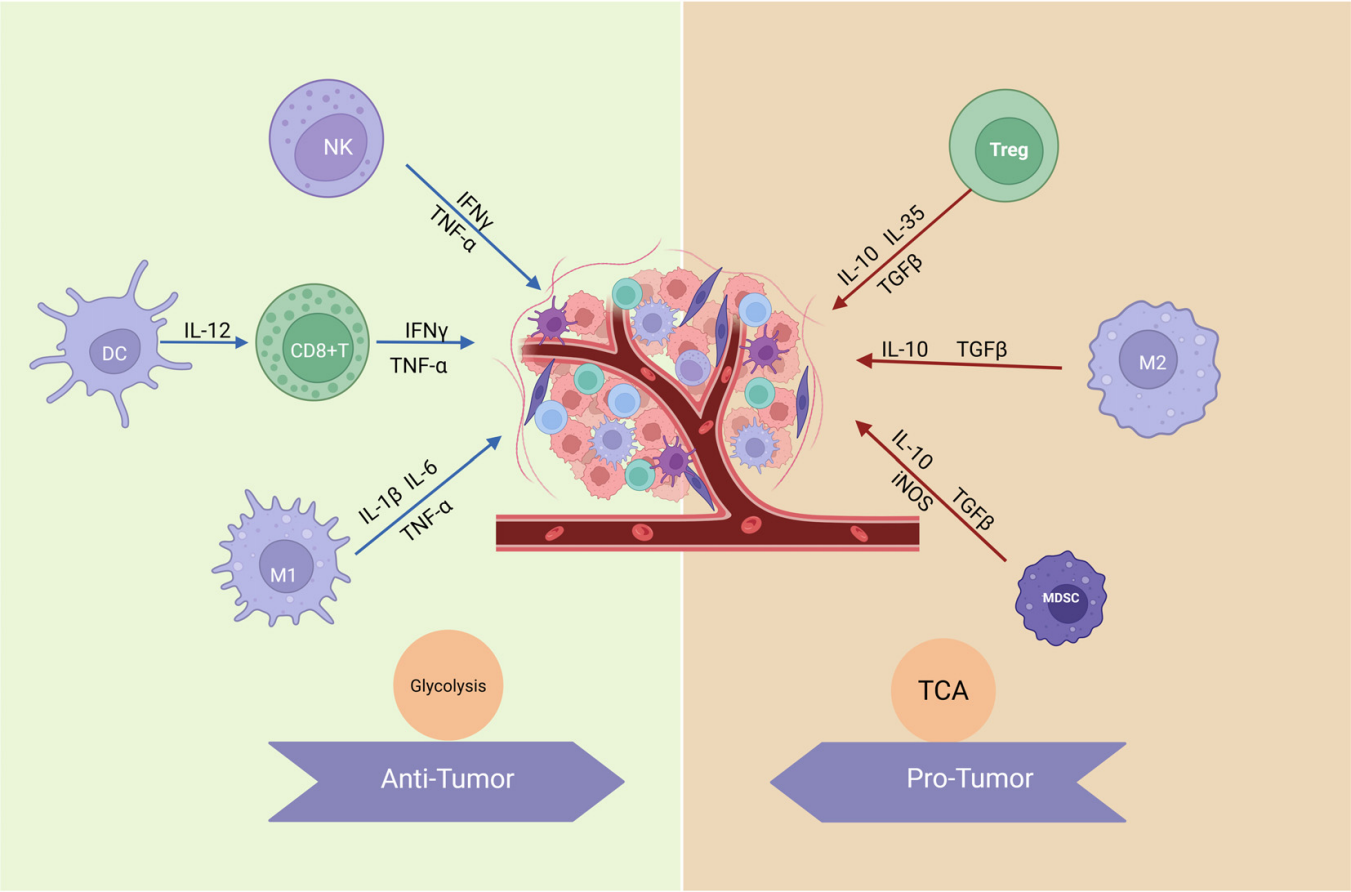
Metabolic mechanisms and functions of immune cells. Anti-tumor immune cells, such as effector T cells (including CD8+ T cells), NK cells, and M1-like macrophages, primarily rely on glycolysis for rapid energy supply to meet their high activity and quick response demands. Glycolysis not only supports ATP production but also provides intermediates for biosynthesis, enabling the secretion of pro-inflammatory cytokines like IFN-γ, IL-1β, and IL-6, which are crucial for tumor elimination and immune activation. In contrast, pro-tumor immune cells, including Tregs and M2-like macrophages, utilize OXPHOS as their dominant metabolic pathway. OXPHOS efficiently generates energy to sustain long-term immunosuppressive functions, facilitating the production of anti-inflammatory cytokines such as IL-10, TGF-β, and IL-35, which promote tumor progression and immune evasion. These metabolic adaptations reflect the functional specialization of immune cells within the TME. (Created with BioRender.com).

The tumor microenvironment (TME) is a dynamic and metabolically distinct ecosystem comprising cancer cells, stromal cells, immune cells, extracellular matrix (ECM), and signaling molecules that collectively drive tumor progression and therapy resistance (1). Hypoxia, a hallmark of the TME, stabilizes hypoxia-inducible factors (HIFs), promoting angiogenesis and metabolic reprogramming to sustain tumor growth (2, 3). These metabolic adaptations are further complemented by immune evasion strategies, including the suppression of cytotoxic T cell activity and the polarization of tumor-associated macrophages (TAMs) toward an immunosuppressive phenotype (4). The metabolic mechanisms and functions of immune cells are illustrated in **Figure 1**. Meanwhile, cancer-associated fibroblasts (CAFs) drive ECM remodeling, promoting tumor invasion and metastasis by interacting with cellular metabolism (5, 6).

Central to these processes is the metabolic byproduct lactate, which accumulates in the TME due to the elevated glycolytic activity of cancer cells—a hallmark of the Warburg effect (7, 8). Beyond serving as a metabolic substrate, lactate acts as a potent signaling molecule, activating pathways such as HIF-1α, TGF-β, and NF-κB to promote angiogenesis, immune evasion, and ECM remodeling (9–11). The acidic microenvironment created by lactate accumulation inhibits the function of cytotoxic T cells and natural killer cells, thereby reinforcing immune suppression (12, 13). Simultaneously, lactate reprograms TAMs, shifting their balance from a pro-inflammatory M1 phenotype to an immunosuppressive, tumor-supportive M2 phenotype (14).

Macrophages are pivotal regulators within the TME due to their functional plasticity. Rather than being strictly categorized as M1 or M2, macrophages exist along a functional spectrum, with TAMs predominantly skewed toward an M2-like phenotype. These TAMs drive tumor progression through angiogenesis, immune suppression, and ECM remodeling (15–17). This reprogramming is mediated by Th2-skewing cytokines (e.g., IL-4, TGF-β1) and tumor-derived growth factors (e.g., CSF1, GM-CSF), fostering a microenvironment that supports tumor growth and metastasis (18). Moreover, TAMs suppress T cell activity by recruiting regulatory T cells via chemokines, inducing Foxp3+ iTreg cells through IL-10 and TGF-β, and depleting L-arginine via arginase I, which impairs TCR signaling (18). At one end of the spectrum, M1-like macrophages exhibit anti-tumor activity through mechanisms such as reactive oxygen species (ROS) and nitric oxide (NO) production or antibody-dependent cell-mediated cytotoxicity (ADCC) (19). However, during tumor progression, macrophages are dynamically reprogrammed by various factors in the TME, gradually shifting toward an M2-like phenotype, which perpetuates an immunosuppressive cycle and accelerates tumor development (20).

Lactate’s role in TAM reprogramming extends beyond its metabolic effects. Lactate drives histone and protein lactylation, an emerging post-translational modification that epigenetically reshapes TAM gene expression, enhancing their pro-tumoral and immunosuppressive functions (21, 22). For instance, lactate-induced lactylation of histones specifically activates genes associated with tissue repair and immunosuppression, reinforcing the M2-like phenotype (22). Concurrently, metabolic reprogramming of TAMs, orchestrated by lactate, promotes reliance on oxidative phosphorylation (OXPHOS) and the tricarboxylic acid (TCA) cycle, hallmarks of M2-like macrophage metabolism (9, 14, 23, 24). Given its multifaceted role in the TME, lactate represents an attractive therapeutic target for cancer treatment. Inhibitors targeting lactate dehydrogenase A (LDHA), a key enzyme in lactate production, and monocarboxylate transporters (MCTs), which mediate lactate transport, have shown promise in shifting TAMs along the functional spectrum from an immunosuppressive M2-like state toward a more pro-inflammatory M1-like state (25, 26). This reprogramming restores anti-tumor immunity and disrupts the immunosuppressive environment of the TME. Furthermore, combining lactate-targeting strategies with immunotherapies, such as PD-1/PD-L1 inhibitors, has demonstrated synergistic potential in preclinical studies by enhancing T cell infiltration and activity (27, 28).

This review explores the critical role of lactate in TAM polarization and tumor progression, highlighting its central position in the metabolic and immune landscape of the TME. Targeting lactate metabolism, particularly in conjunction with established immunotherapies, represents a promising strategy to overcome immune suppression, improve therapeutic outcomes, and ultimately reshape the TME for effective cancer treatment.

## 2 Lactate metabolism in the tumor microenvironment

### 2.1 Lactate production metabolism

Lactate production in the TME is primarily driven by two metabolic pathways, glycolysis and glutaminolysis, which are critical for sustaining tumor growth and progression (29). Tumor cells predominantly rely on aerobic glycolysis, known as the “Warburg effect,” converting glucose to lactate via LDHA, even in normoxic conditions (29). This process regenerates NAD^+^;, maintaining glycolytic flux and supporting rapid proliferation (30). The rate of glycolysis is tightly regulated by glycolytic enzymes through allosteric modulation, oncogenic signaling-driven expression changes, and post-translational modifications affecting activity, localization, and stability (31). HIF-1α plays a central role in regulating glycolysis and lactate production under hypoxic conditions by upregulating glycolytic genes such as Glut1, HK2, LDHA, and PKM2 (32). Hypoxia further enhances lactate production; however, knockout of HIF-1α significantly reduces extracellular acidification and abolishes hypoxia-induced lactate accumulation, highlighting its critical role in metabolic adaptation (30, 32).

**Figure 2:**
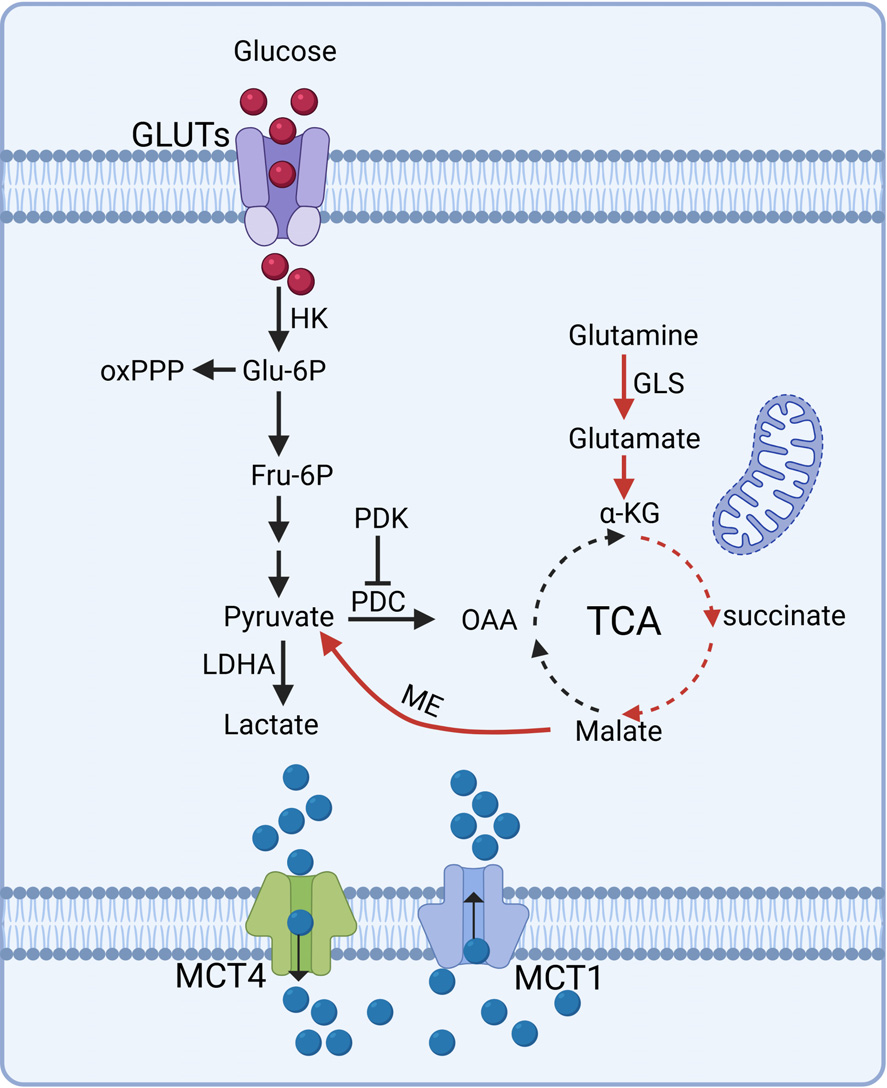
Molecular mechanisms of intracellular lactate production and export. Tumor cells predominantly rely on aerobic glycolysis, converting glucose into lactate via LDHA even in normoxic conditions, sustaining glycolytic flux and rapid proliferation. Glutaminolysis serves as an alternative pathway, where glutamine is metabolized into α-KG, feeding the TCA cycle and contributing to lactate production. Lactate transport is mediated by MCTs: MCT1 facilitates lactate influx or efflux depending on tissue type, while MCT4 predominantly drives lactate efflux in glycolytic or hypoxic cells. These pathways underscore tumor cells' metabolic flexibility and reliance on lactate dynamics for energy and biosynthesis. (Created with BioRender.com).

Glutamine serves as a critical energy source in tumor cells, fueling metabolic pathways that contribute to lactate production. In parallel, glutaminolysis acts as an alternative pathway, where glutamine is converted to glutamate by glutaminase (GLS) and subsequently transformed into α-ketoglutarate (α-KG), which feeds into the TCA cycle. Through a series of enzymatic reactions, malate is generated within the cycle, exits the mitochondria, and is converted into pyruvate by malic enzyme. Pyruvate is subsequently converted into lactate, underscoring the metabolic flexibility of tumor cells and their dependence on glutamine for energy production and biosynthetic precursors. This pathway highlights the central role of glutamine metabolism in supporting tumor cell growth, survival, and adaptation under nutrient-limiting conditions (33–35). The molecular mechanisms underlying intracellular lactate production and export are depicted in **Figure 2**.

Besides tumor cells, CAFs and immune cells also contribute to lactate production within the TME. Metabolically reprogrammed CAFs exhibit enhanced glycolysis, generating lactate to support tumor growth (29). Similarly, immune cells such as macrophages and T cells undergo glycolytic reprogramming in the TME, further exacerbating lactate accumulation and shaping the metabolic landscape of the TME (14, 36–38).

### 2.2 Lactate secretion mechanism

In tumor cells, lactate transport is predominantly regulated by monocarboxylate transporters (MCTs), which are encoded by the SLC16 gene family (39). MCT1 is ubiquitously expressed, supporting lactate influx in oxidative tissues (e.g., heart, red muscle) and efflux in glycolytic cells. MCT2 is tissue-specific, found in liver, kidney, and neurons, facilitating lactate-driven gluconeogenesis and mitochondrial respiration. MCT3 is exclusively expressed in the eye, regulating subretinal pH. MCT4, with the lowest lactate affinity, primarily enables lactate efflux in glycolytic or hypoxic cells (e.g., white muscle, astrocytes), supporting metabolic exchanges between lactate-producing and -consuming cells, such as in skeletal muscles and the central nervous system (40). Among the MCT family, MCT1 and MCT4 (encoded by SLC16A1 and SLC16A3, respectively) are most relevant in cancer, facilitating lactate metabolism and pH homeostasis. Both are frequently upregulated in tumors such as brain, breast, cervix, and colorectal cancers, promoting progression (39–41). MCT1 is also essential for macrophage polarization and function. Knockdown of MCT1 significantly diminishes M2 macrophage polarization markers (42). MCTs facilitate the export of lactate and protons from glycolytic tumor cells and the import of lactate into oxidative tissues, supporting metabolic adaptation in tumors (41, 43).

MCT4 is predominantly upregulated under hypoxic conditions via HIF-1α, while MCT1 expression is driven by MYC, Nrf2 and epigenetic modifications (41, 43). These transporters enable lactate efflux, which creates an acidic microenvironment and promotes metabolic reprogramming essential for tumor growth and survival. Targeting MCT1 and MCT4 with small-molecule inhibitors has shown promise in disrupting tumor metabolism, though dual inhibition is required to effectively block lactate-driven tumor progression in hypoxic conditions (41, 44).

The functionality of MCT1 and MCT4 depends on their interaction with CD147 (Basigin), a transmembrane glycoprotein critical for MCT membrane localization (41, 45). CD147 binds MCT monomers through its transmembrane domain, with Glu218 serving as a key binding site (46, 47). This interaction is essential for glycolysis, ATP production, and tumor cell proliferation (45, 46). In CD147-deficient mice, the absence of MCT expression in retinal cells disrupts lactate transport, leading to energy depletion and visual impairment (45, 48). The interplay between MCTs and CD147 is fundamental to lactate metabolism and tumor adaptation, emphasizing the significance of lactate transport in cancer progression.

## 3 Functional polarization and metabolic reprogramming of macrophages within the tumor microenvironment

Macrophages exhibit a dynamic spectrum of phenotypes, ranging from M1 macrophages, to anti-inflammatory M2 macrophages, including subtypes M2a, M2b, and M2c. Intermediate phenotypes, such as the transitional M0 state, further enrich this continuum (49). Microenvironmental signals dynamically regulate macrophage polarization, driving gradual transitions across this functional and metabolic continuum (50). Classically activated M1 macrophages are skewed toward anti-tumor immune responses. Induced by lipopolysaccharide (LPS) and interferon-gamma (IFN-γ), M1 macrophages recognize tumor cells through surface antigens and execute their functions by producing reactive oxygen species (ROS), inducible nitric oxide synthase (iNOS) coupled with the production of pro-inflammatory cytokines (51–53). They secrete key inflammatory mediators such as interleukin-1β (IL-1β), interleukin-6 (IL-6), TNF and interleukin-12 (IL-12), driving strong pro-inflammatory responses to combat tumors (53). In contrast, alternatively activated M2 macrophages, stimulated by IL-4, IL-10, or IL-13, perform immunosuppressive and tissue-repair roles (53, 54). M2 macrophages are anti-inflammatory, characterized by low antigen-presentation capability, reduced IL-12 secretion, and high production of IL-10, IL-4, and arginase-1 (Arg-1), which suppress inflammation and promote tissue remodeling (53, 54). Their functional diversity is reflected in subtypes including M2a, M2b, and M2c, which contribute to immune regulation and homeostasis (52, 55). M1 and M2 macrophages contribute complementarily to immune balance and the response to pathological conditions. Notably, these macrophage states form a functional continuum rather than distinct, opposing categories.

The functional divergence between M1 and M2 macrophages is intricately linked to their metabolic reprogramming. Typically, M1 macrophages undergo a metabolic transition from oxidative phosphorylation (OXPHOS) to glycolysis, accompanied by an upregulated pentose phosphate pathway (56). This metabolic reprogramming leads to the accumulation of key metabolites, including succinate, citrate, and itaconate, while simultaneously disrupting the TCA cycle (57). Succinate stabilizes HIF-1α, promoting glycolytic gene transcription, while citrate and itaconate contribute to the production of inflammatory mediators such as IL-1β, nitric oxide, and prostaglandins (53, 58). The pentose phosphate pathway (PPP), plays a crucial role in cytokine production, redox homeostasis, and biosynthesis, supporting M1-driven inflammation (57, 59). Conversely, M2 macrophages rely on an intact TCA cycle to meet ATP demands for UDP-GlcNAc-dependent glycosylation, which is critical for lectin and mannose receptor functions (56, 57). Their metabolism is dominated by OXPHOS and FAO, with the TCA cycle and glutaminolysis providing substrates for the electron transport chain and sustaining oxygen consumption (57). Glutamine metabolism is essential in M2 macrophages, as it replenishes TCA cycle intermediates and provides nitrogen for UDP-GlcNAc synthesis (56). Glycolipid metabolism further regulates M2 polarization, while mitochondrial biogenesis, marked by increased expression of mitochondrial transcription factor A (TFAM) and cytochrome c oxidase subunit 1 (Cox-1), enhances their ability to mediate anti-inflammatory responses and tissue repair (53, 58). This metabolic flexibility highlights the continuous spectrum of macrophage polarization, driven by intrinsic and extrinsic signals, including lactate and tumor-derived metabolites. Lactate plays a central role in shaping macrophage phenotypes by modulating metabolic pathways and cellular signaling, thereby influencing immune responses. Understanding these mechanisms provides critical insights into immune regulation and offers promising opportunities for therapeutic interventions, particularly in cancer treatment.

Given the distinct metabolic requirements of M1 and M2 macrophages, targeting their metabolism offers a promising strategy to regulate polarization. Inhibiting glycolysis, either by activating pyruvate kinase M2 (PKM2) or suppressing pyruvate dehydrogenase kinase 1 (PDK1), has been shown to impair M1 polarization (58, 60, 61). Similarly, silencing CXCR4 inhibits glycolysis and facilitates M2 to M1 transition, highlighting a potential metabolic switch (62). In tumor-bearing mice, combining the glycolysis inhibitor 2-DG with radiation therapy restored M1 phenotypes and reduced tumor burden, demonstrating the therapeutic potential of metabolic reprogramming in the TME (63).

## 4 Effects of lactate on macrophage metabolic reprogramming

**Figure 3:**
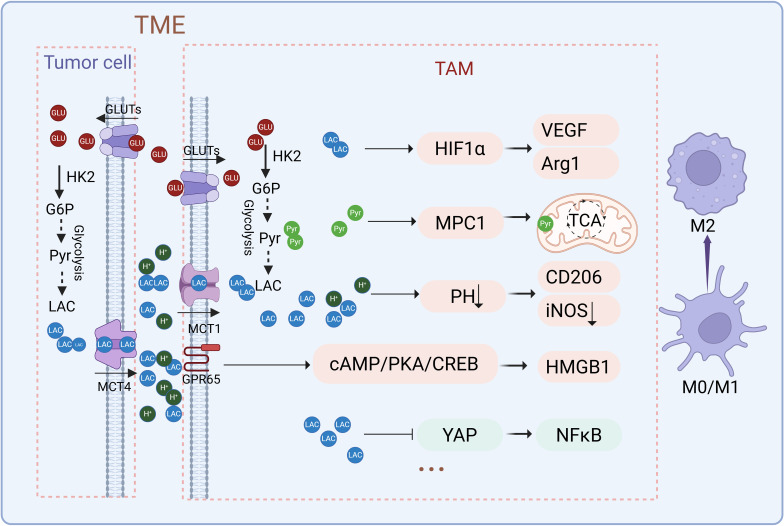
Effects and mechanisms of lactate on macrophage polarization. Lactate regulates macrophage polarization through multiple mechanisms, including the stabilization of HIF-1α, modulation of NF-κB signaling, activation of lactate receptors GPR65, and pH-dependent effects. These pathways converge to upregulate key factors such as Arg1, VEGF, and CD206, collectively driving the transition toward an immunosuppressive M2 phenotype. (Created with BioRender.com).

Lactate plays a dynamic and context-dependent role in macrophage polarization, influencing the balance along the functional spectrum between pro-inflammatory M1 and anti-inflammatory M2 phenotypes (25, 64). The effects and mechanisms of lactate on macrophage polarization are illustrated in **Figure 3**. High concentrations of lactate in macrophages treated with LPS induce a time- and dose-dependent shift from M1 to M2 polarization, as indicated by elongated cell morphology, increased Arg1 expression, and reduced CD86 and iNOS expression (65). Overexpression of LDHA or supplementation with exogenous lactate further promotes M2 polarization by upregulating markers like IL-10 and Arg1 while suppressing M1 markers such as TNF and IFN-γ (25). Conversely, deleting LDHA in macrophages drives M1 polarization, suppresses VEGF expression and angiogenesis, and enhances effector CD8+ T-cell activity (66). Targeting lactate metabolism through the inhibition of lactate efflux reduces M2 macrophage populations, increases CD8+ T cell infiltration, and improves the efficacy of anti-PD1 therapy in pancreatic tumors (67). Interestingly, in bone marrow-derived macrophages (BMDMs) treated with IL-4 or IFN-γ/LPS, lactate suppresses M2-associated genes (Arg1, Cd163, Cd206, Il-10) and markers (e.g., Arg1, IL-10), while enhancing M1-associated genes (Nos, TNF, IL-12) and markers (e.g., TNF, iNOS) (24). This switch decreases CD206^+^; F4/80^+^; M2 macrophages while increasing CD86^+^; F4/80^+^; M1 macrophages, accompanied by distinct morphological changes (24).

Lactate export from tumor cells is proton-coupled, leading to its accumulation alongside intratumoral acidification. This acidification alters the microenvironment’s pH, favoring distinct macrophage phenotypes: a neutral pH (7.4) tends to influence macrophages toward a more pro-inflammatory, M1-like state with elevated iNOS expression, while an acidic pH (6.7) favors an anti-inflammatory, M2-like functional state, characterized by increased CD206 expression and reduced iNOS levels (64). TAMs sense tumor-derived lactate and microenvironment acidification through GPR132 and GPR65 in a non-redundant manner, driving M2 polarization (68). Lactate activates GPR65 on TAMs, triggering the cAMP-PKA-CREB signaling cascade to promote HMGB1 secretion, thereby facilitating glioma progression (69). Melanomas, with heightened glycolytic activity, produce excessive lactate, leading to significant TME acidification. This acidic environment predominantly drives TAMs toward M2-like polarization via GPR65, upregulating key genes such as Arg1 and VEGF. Notably, knocking out GPR65 abolishes this effect, highlighting its critical role in macrophage regulation (70). Additionally, studies have shown that an acidic pH (6.8), even independent of lactate, can induce anti-inflammatory macrophage polarization by upregulating markers like Arg1 and CD206 while suppressing pro-inflammatory markers such as NOS2 and IL-6. Neutralizing tumor acidity has been demonstrated to mitigate the pro-tumor phenotypes of TAMs, emphasizing the pivotal role of pH in macrophage polarization and tumor progression (71).

Lactate exerts its regulatory effects on macrophages through distinct signaling pathways, including HIF-1α and NF-κB (72, 73). Tumor-derived lactate, absorbed by macrophages through monocarboxylate transporters (MCT1–4), activates HIF-1α, which subsequently upregulates the expression of vascular endothelial growth factor (VEGF) and arginase-1 (Arg1) (73). This activation promotes an immunosuppressive, M2-like phenotype that supports tumor progression. Inhibiting MCTs blocks lactate uptake and downstream signaling, while genetic deletion of HIF-1α abolishes lactate-induced polarization, underscoring HIF-1α’s central role in lactate-driven metabolic reprogramming (73). In addition to HIF-1α, lactate regulates macrophage polarization through the NF-κB pathway. NF-κB plays a crucial role in M1 polarization by promoting the synthesis of pro-inflammatory cytokines (72). LPS stimulation enhances NF-κB activation via interactions with Yes-associated protein (YAP) and the NF-κB subunit p65, amplifying inflammatory responses (72). However, lactate suppresses this interaction by inhibiting YAP activation, reducing NF-κB nuclear translocation, and lowering pro-inflammatory cytokine production. This inhibition dampens M1 polarization while promoting an immunosuppressive environment (72). Elevated lactate levels also enhance macrophage polarization by activating NF-κB, leading to increased PD-L1 expression, which facilitates immune evasion and tumor progression (74).

Lactate supports mitochondrial oxidative metabolism in IL-4-induced M2 macrophages by serving as a substrate for the TCA cycle (23). Lactate, absorbed by macrophages and converted to pyruvate, enters the TCA cycle through mitochondrial pyruvate carrier 1 (MPC1) (23). Blocking MPC1 significantly reduces IL-4-induced M2 polarization and associated gene expression, highlighting the dependence of M2 macrophages on mitochondrial metabolism (23). Both glucose- and lactate-derived mitochondrial pyruvate are critical for M2 polarization and their immunosuppressive functions, including suppressing CD8^+^; T cell proliferation and IFN-γ production (23). TCA cycle intermediates also influence macrophage polarization. Succinate, elevated in macrophages upon LPS stimulation, stabilizes HIF-1α and promotes IL-1β production by inhibiting prolyl hydroxylase (PHD). This stabilization enhances M1 polarization and inflammatory responses, contrasting with lactate’s effects on M2 polarization (57, 74).

In summary, lactate has a complex role in macrophage polarization, influencing processes such as altering microenvironmental pH, activating key signaling pathways like HIF-1α and NF-κB, and supporting mitochondrial metabolism in M2 macrophages. It primarily fosters an immunosuppressive phenotype, which in turn promotes tumor progression. However, its effects are dynamic and context-dependent. As a crucial factor in the TME, further investigation into lactate’s mechanisms could provide valuable insights and reveal new therapeutic targets for cancer treatment.

## 5 Lactate regulates macrophage polarization through lactylation modification

### 5.1 Histone lactylation

Histone modifications are critical regulators of chromatin structure, gene expression, DNA replication, and repair, thereby maintaining cellular homeostasis and genomic stability (21). Among these, histone lactylation (Kla) has emerged as a novel and significant epigenetic modification influencing macrophage polarization and immune responses. In 2019, Zhao et al. discovered lysine lactylation (Kla) as a distinct histone modification originating from lactate. Through HPLC-MS/MS, synthetic peptides, pan anti-Kla antibodies, and isotopic labeling, they validated the lactate-derived nature of Kla and identified numerous Kla sites in human MCF-7 cells and mouse macrophages (22). During M1 macrophage polarization, elevated lactate production via aerobic glycolysis correlates with increased histone Kla levels and decreased histone acetylation (Kac). Notably, histone Kla specifically promotes the late-phase expression of M2-like homeostatic genes, such as Arg1, without affecting early pro-inflammatory gene expression. This epigenetic regulation is mediated by p300, which acts as a potential Kla writer (22). Additionally, MCT4 deficiency enhances H3K18la lactylation at reparative gene loci (e.g., IL-10, PDHA1), promoting their transcription. Unlike other modifications such as methylation or acetylation, lactylation dominates during the transition to M2 macrophages, linking metabolic shifts to epigenetic remodeling and macrophage function (75). Furthermore, lactate enhances CCL18 expression in macrophages through GPR132-mediated H3K18 lactylation, selectively inducing the transcriptional activation of the CCL18 promoter, thereby linking histone lactylation to macrophage-mediated immune responses (76).

Therapeutically, targeting histone lactylation has shown promise in modulating macrophage polarization. For instance, VB124 and MD-43, inhibitors of monocarboxylate transporter 4 (MCT4), enhance H3K18la levels through p300, driving macrophages toward a reparative phenotype characterized by increased anti-inflammatory and TCA cycle gene expression and reduced pro-inflammatory and glycolysis gene expression (75). These compounds effectively decrease macrophage content in plaques and slow atherosclerosis progression in high-fat diet-fed ApoeKO mice. MD-43, in particular, selectively degrades MCT4 via the UPS pathway, further amplifying H3K18la enrichment at reparative gene promoters, highlighting its therapeutic potential (75). Additionally, mitochondrial fragmentation in macrophages during inflammatory activation enhances lactate production and histone lactylation, driven by decreased PDH expression and increased pyruvate-to-lactate conversion. This metabolic shift promotes the expression of reparative genes like Arg1 via histone lactylation, linking mitochondrial dynamics to macrophage polarization and tissue repair (77). Dysregulated glycolysis and MCT1-mediated lactate transport further regulate histone lactylation through IL-1β-dependent recruitment of GCN5, enhancing the transcription of reparative genes such as Lrg1, Vegf-a, and IL-10, which foster anti-inflammatory responses and pro-angiogenic activities (78).

### 5.2 Non-histone lactylation

Besides histones, lactylation also occurs on non-histone proteins, underscoring its broader regulatory roles in cellular functions such as gene expression, DNA repair, cell cycle regulation, signaling, and metabolism (21). Recent studies have uncovered its critical roles in diverse biological processes, including macrophage polarization and protozoan metabolism. For example, exercise-induced lactylation of methyl-CpG-binding protein 2 (MeCP2) at K271 promotes M2 macrophage polarization, enhances plaque stability, and suppresses RUNX1 transcription, showcasing the therapeutic potential of lactylation in treating atherosclerosis (79). Similarly, in *Trypanosoma brucei*, lysine lactylation extends beyond histones, with 387 sites identified in 257 non-histone proteins. Regulated by glucose metabolism through a unique lactate pathway, non-histone lactylation in *T. brucei* plays essential roles in energy metabolism and gene expression, uncovering novel regulatory mechanisms in early-branching eukaryotes (80).

**Figure 4:**
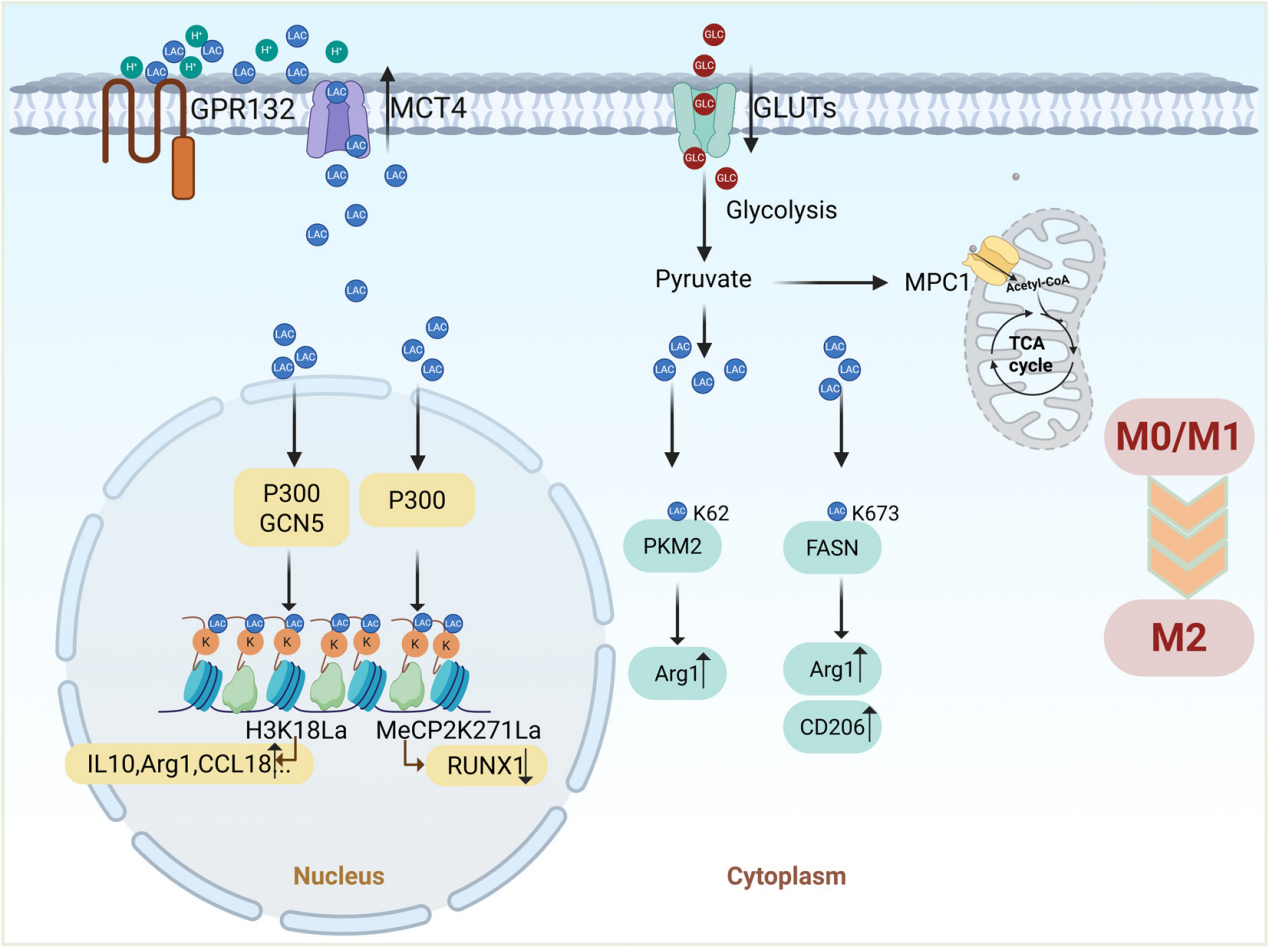
Lactate regulates macrophage polarization through lactylation modification. Lactate-driven lactylation connects metabolism to macrophage polarization by modifying histones, non-histone proteins, and metabolic enzymes. Histone lactylation (Kla) promotes the expression of M2-related genes (e.g., Arg1), while non-histone lactylation (e.g., MeCP2) and modifications of metabolic enzymes (e.g., PKM2, FASN) further facilitate the transition to an anti-inflammatory M2 phenotype. (Created with BioRender.com).

Lactylation also regulates key metabolic enzymes. Lactate promotes the K62 lactylation of pyruvate kinase M2 (PKM2), enhancing its activity and driving the transition of LPS-induced macrophages from a pro-inflammatory state to a reparative phenotype. The K62R mutant of PKM2 abolishes lactate’s regulatory effects on both PKM2 activity and macrophage phenotype transition, underscoring the essential role of K62 lactylation in these processes (81). Similarly, inhibition of mitochondrial pyruvate carrier 1 (MPC1) prevents pyruvate entry into mitochondria, leading to elevated lactate production and increased FASN K673 lactylation. This modification reduces lipid accumulation in hepatocytes, suppresses inflammatory cytokine expression, and facilitates M2 macrophage polarization, fostering an anti-inflammatory environment (82). **Figure 4** illustrates how lactate regulates macrophage polarization through lactylation modification, highlighting a key regulatory mechanism.

## 6 Targeting lactate and lactylation in macrophage metabolic reprogramming for cancer therapy

### 6.1 Therapeutic potential of targeting lactate

Lactate is crucial in driving tumor progression by promoting immune suppression and reshaping the TME. Tumor-derived lactate, transported into macrophages via MCT1, not only stabilizes HIF-1α but also induces histone lactylation, facilitating macrophage polarization independently of endogenous lactate or MPC-mediated metabolism (83). Beyond macrophage polarization, lactate suppresses T cell proliferation, cytokine production, and cytotoxicity, while inducing apoptosis and inhibiting NK cell and type 2 innate lymphoid cells (ILC2) activity through intracellular acidification (36, 83). Moreover, histone lactylation caused by lactate recruits CAFs, TIMs, and CSCs, further remodeling the TME to support tumor progression and metastasis (84). In prostate cancer, lactate activates KIAA1199 via HIF-1α-mediated lactylation, driving angiogenesis, vasculogenic mimicry, and hyaluronic acid depolymerization, ultimately accelerating tumor progression (85). Importantly, lactate-induced macrophage polarization and dynamic changes in the TME are interdependent, forming a positive feedback loop that synergistically fosters immune suppression and tumor progression. These multifaceted roles highlight lactate’s therapeutic potential. By targeting key metabolic enzymes (e.g., LDHA, HK2) or transporters (e.g., MCTs, GLUTs), lactate production and transport can be disrupted, reprogramming macrophages and alleviating immune suppression to enhance antitumor immunity. **Table 1** provides a comprehensive summary of lactate-associated therapeutic targets and their corresponding inhibitors in cancer therapy.

**Table 1:** Lactate-associated targets and inhibitors in cancer therapy.

Target	Inhibitor	Cancer Types	Clinical Trials	Reference
LDHA	FX11	Pancreatic cancer, Prostate cancer	Preclinical studies	(116, 123)
LDHA	GNE-140	Breast cancer, Melanoma	Preclinical studies	(124, 125)
LDHA	Galloflavin	Endometrial cancer, Colorectal cancer	Preclinical studies	(126–128)
LDHA	Oxamate	Gastric cancer, Glioblastoma	Preclinical studies	(129, 130)
HKII	3-Bromopyruvate	Colorectal cancer	Preclinical studies	(87)
PDK	Dichloroacetate (DCA)	Glioblastoma, Breast cancer	Phase I/II clinical trials (e.g., NCT05120284)	(131, 132)
Mitochondrial electron transport chain	Atovaquone	Breast cancer, Non-small cell lung cancer	Phase II clinical trial (NCT02628080)	(133–135)
Hexokinase, MCTs	Lonidamine	Non-small cell lung cancer, Breast cancer	Phase II/III clinical trials (e.g., NCT03718767)	(136)
MCT1	AZD3965	Lymphoma, Small cell lung cancer, Colorectal adenocarcinoma	Phase I clinical trial (NCT01791595)	(137–139)
MCT1	7ACC2	Breast cancer, Squamous cell carcinoma, Pancreatic adenocarcinoma	Preclinical studies	(140, 141)
MCT1	SR13800	Ovarian cancer, Neuroblastoma	Preclinical studies	(142, 143)
MCT4	VB124	Hepatocellular carcinoma	Preclinical studies	(95)
MCT4	AZD0095	Non-small cell lung cancer	Preclinical studies	(144)
MCT1 MCT4	syrosingopine	Leukemia, Breast cancer	Preclinical studies	(44)
GLUT1 GLUT4	WZB117	Lung cancer, Breast cancer	Preclinical studies	(98, 99)
GLUT1	BAY-876	Head and neck squamous Carcinoma	Preclinical studies	(100)
ALDH1A3	D34-919	Glioblastoma	Preclinical studies	(109)
AARS1	β-Alanine	Colorectal carcinoma	Preclinical studies	(110)

#### 6.1.1 Hexokinase 2

HK2 catalyzes the first committed step of glycolysis, phosphorylating glucose to glucose-6-phosphate (86). In cancer cells, HK2 is often overexpressed, driving the Warburg effect by promoting elevated glycolytic flux and lactate production even under aerobic conditions (86). This metabolic reprogramming supports tumor growth by providing energy, biosynthetic precursors, and an acidic microenvironment that suppresses immune responses. Targeting HK2 has shown significant promise in disrupting cancer metabolism. HK2 inhibitors reduce glycolysis, lower lactate production, and normalize the TME by decreasing acidity and promoting immune activation (86, 87). Preclinical studies report that HK2 inhibition not only impairs tumor growth but also enhances TAM polarization toward the M1 phenotype, boosting anti-tumor immunity. Furthermore, combining HK2 inhibitors with other therapies, such as immune checkpoint inhibitors, offers synergistic benefits, amplifying therapeutic efficacy by exploiting cancer-specific metabolic vulnerabilities (87).

#### 6.1.2 Lactate dehydrogenase A

LDHA, a central enzyme in lactate metabolism, facilitates the conversion of pyruvate to lactate while regenerating NAD^+^;, a crucial cofactor for glycolysis (88). Although LDHA activity is typically elevated under hypoxic conditions, cancer cells exhibit aberrant overexpression of LDHA even in normoxic environments (89). This metabolic shift promotes excessive lactate accumulation, which acidifies the TME, suppresses anti-tumor immune responses, and fosters angiogenesis, ECM remodeling, and metastasis (88, 90, 91). Importantly, LDHA activity directly influences TAM polarization within the TME. High lactate levels favor M2-like polarization, linking LDHA overexpression to both metabolic and immune suppression in cancer. Clinically, LDHA represents a compelling therapeutic target. LDHA inhibition reduces lactate production, alleviates TME acidification, and reprograms TAMs toward a pro-inflammatory M1 phenotype. This shift boosts anti-tumor immunity and suppresses tumor progression (25). Preclinical studies have demonstrated that LDHA inhibitors effectively reduce tumor growth, metastasis, and therapy resistance, making LDHA a central nexus in cancer metabolism and immune modulation (12, 92–94).

#### 6.1.3 Monocarboxylate transporters

MCTs, particularly MCT1 and MCT4, are integral to lactate transport in cancer cells. MCT1 facilitates lactate and pyruvate uptake, while MCT4 mediates lactate export, maintaining intracellular pH homeostasis and sustaining glycolytic flux (95). In the TME, these transporters enable cancer cells to efficiently shuttle lactate, creating an acidic microenvironment that promotes immune evasion, angiogenesis, and tumor progression.

Targeting MCTs represents a promising strategy to disrupt lactate dynamics in tumors. Inhibitors such as AZD3965 (MCT1-specific) and syrosingopine (dual MCT1/MCT4) block lactate transport, inducing metabolic stress and enhancing the efficacy of immunotherapies (44, 95, 96). Preclinical studies have demonstrated the potential of MCT inhibitors in reducing tumor growth and sensitizing cancer cells to existing treatments (96). However, challenges like tumor heterogeneity and compensatory metabolic pathways emphasize the need for combination therapies to maximize the therapeutic impact of MCT inhibition.

#### 6.1.4 Glucose Transporters

GLUTs, especially GLUT1, are crucial regulators of glucose uptake in cancer cells, fueling glycolysis and subsequent lactate production (97). Overexpression of GLUT1 and other isoforms, such as GLUT3, is a common feature in tumors, supporting elevated glycolytic flux to provide ATP and biosynthetic precursors for rapid cell proliferation (97, 98). Lactate accumulation driven by GLUT-mediated glycolysis further contributes to immune evasion and metastatic potential by creating an acidic TME. Therapeutically, GLUT inhibition offers a viable approach to target tumor metabolism. Small-molecule inhibitors such as WZB117 and BAY-876 effectively reduce glucose uptake, suppress glycolysis, and impair tumor growth in preclinical models (98–100). Moreover, GLUT inhibition has been shown to enhance the efficacy of chemotherapy, radiotherapy, and immunotherapy by disrupting cancer cell metabolic flexibility (100, 101). These findings indicate the potential of GLUT inhibitors as a complementary strategy in cancer treatment.

#### 6.1.5 Pyruvate dehydrogenase kinases

Pyruvate dehydrogenase kinases (PDKs) are key regulators of the conversion of pyruvate to acetyl-CoA by modulating the activity of the pyruvate dehydrogenase complex (PDC), thereby governing its entry into the TCA cycle (102, 103). By phosphorylating and inactivating PDC, PDKs shift metabolism toward glycolysis. The overexpression of PDKs, especially PDK1 and PDK3, has been linked to unfavorable clinical outcomes and resistance to therapeutic interventions across multiple cancer types. PDKs also play a significant role in macrophage metabolism (61, 104). M1 macrophages exhibit glycolytic reprogramming similar to the Warburg effect, with PDK1 essential for the expression of M1-specific markers (22, 105). Pharmacological inhibition of PDKs, such as with dichloroacetate (DCA), restores PDC activity, redirects pyruvate metabolism toward oxidative phosphorylation, and reduces glycolytic flux. This metabolic reprogramming triggers pyroptosis in tumor cells and potentiates the effectiveness of immunotherapeutic approaches (106, 107). These findings position PDK inhibitors as promising agents to modulate both tumor metabolism and immune cell function.

### 6.2 Therapeutic potential of targeting lactylation

In the process of macrophage polarization, lactylation shows the ability to balance inflammatory reactions and tissue regeneration, exhibiting dual functions. In M1 macrophages, increased histone lactylation during the late polarization phase activates homeostatic genes essential for wound healing, contributing to immune homeostasis and tissue regeneration (22). This dual regulatory function highlights the therapeutic potential of targeting lactylation to modulate macrophage activity, offering promising avenues for treating inflammatory diseases and cancer.

In the context of cancer, lactylation has been implicated in therapy resistance by regulating DNA repair mechanisms. MRE11, a key protein in homologous recombination (HR), undergoes lactylation at lysine 673 (K673), a modification mediated by CBP acetyltransferase and regulated by ATM. Lactylation enhances MRE11’s DNA-binding ability, facilitating HR-mediated DNA repair. Inhibiting CBP or LDH reduces MRE11 lactylation, impairs HR activity, and sensitizes tumor cells to cisplatin and PARP inhibitors, demonstrating the therapeutic relevance of targeting lactylation in cancer treatment (108). Similarly, in glioblastoma stem cells, ALDH1A3-driven pyruvate kinase activation promotes XRCC1 lactylation-mediated DNA repair and resistance to chemoradiotherapy. Targeting this pathway with the ALDH1A3 inhibitor D34–919 reverses resistance, underscoring the potential of lactylation-related interventions in glioblastoma therapy (109).

The discovery of lactylation-regulating enzymes, including P300, AARS1, CBP, and YiaC, has deepened our understanding of the functional roles of lactylation (21, 110–112). In macrophages, lactylation influences the expression of profibrotic genes through histone modifications, as evidenced by reduced gene expression following P300 knockdown (113). Beyond macrophages, lactylation can impair tumor suppressor activity, such as the lactylation of p53 mediated by tumor-derived L-lactate via AARS1. This modification weakens p53’s DNA-binding ability and tumor-suppressive functions, while β-alanine prevents p53 lactylation, enhancing chemotherapy efficacy (110). These findings highlight the therapeutic potential of targeting lactylation-regulating enzymes to modulate immune responses and overcome cancer therapy resistance.

In a word, lactylation-regulated macrophage polarization represents a critical mechanism at the intersection of immune modulation and cancer therapy. By influencing macrophage function and mediating tumor resistance, lactylation offers a unique therapeutic target for addressing both tumor progression and treatment challenges. Future efforts should focus on uncovering the molecular mechanisms of lactylation and developing specific inhibitors or activators to exploit its therapeutic potential. Lactylation-based strategies could form the foundation of next-generation cancer therapies, addressing immune dysregulation and overcoming therapy resistance in tandem.

### 6.3 Enhancing immunotherapy via lactate modulation

Lactate metabolism modulation presents a promising strategy to enhance the efficacy of immunotherapy by overcoming therapeutic resistance. By reducing lactate accumulation or inhibiting lactate-mediated signaling pathways, macrophage polarization can be reprogrammed, facilitating the transition from M2-like to M1-like state (64, 65, 72, 73). This shift not only rebalances the TME but also creates a more immunostimulatory milieu that enhances the effectiveness of various immunotherapies, including immune checkpoint inhibitors, such as anti-PD-1 or anti-PD-L1 antibodies (27, 28), CAR-T cell therapy, and cancer vaccines (114). In the TME, tumor-derived exosomes promote the polarization of TAMs towards the M2 phenotype, which is a key mediator of resistance to anti-PD-1 therapy (115). M2 TAMs contribute to immunosuppression and tumor progression mainly through the secretion of cytokines (e.g., TGF-β, PGE2), expression of immune checkpoint ligands (e.g., PD-L1, VISTA), and the release of exosomal microRNAs (e.g., miR-21, miR-155-5p), all of which inhibit the activity of CD8^+^; T cells and limit the anti-tumor immune response (115, 116). These findings suggest the potential of targeting TAMs as a therapeutic strategy to overcome resistance to immunotherapy and improve treatment outcomes. Recent studies demonstrate that combining conventional chemotherapies, such as oxaliplatin and cyclophosphamide, with lactate modulation can further reprogram macrophages, driving M1 polarization and early chemokine upregulation. This reprogramming enhances the inflammatory response and recruits immune cells to the tumor site (114). Additionally, CAR-T cell-derived IFNγ further activates M1-like macrophages expressing iNOS, which produce chemokines such as Cxcl9 and Cxcl10. These chemokines promote CXCR3-dependent immune cell recruitment, establishing a positive feedback loop that amplifies CAR-T cell tumor infiltration and enhances the overall anti-tumor immune response (114).

## 7 Discussion and perspectives

Tumor cells and their microenvironment exhibit profound heterogeneity, a key factor that influences macrophage polarization and contributes to therapeutic resistance across various cancer types. In prostate cancer, docetaxel-treated cells secrete CSF-1 to recruit TAMs, which then activate CXCR4 signaling via CXCL12 secretion, promoting tumor cell survival and resistance to chemotherapy (117). In breast cancer, chemotherapy-induced tumor debris triggers HO-1 upregulation in TAMs, suppressing M1 polarization and contributing to an immunosuppressive microenvironment (118). In lung cancer, Oct4 expression enhances M-CSF secretion, driving TAMs toward an M2 phenotype that supports tumor progression (119). These findings highlight the significant heterogeneity of TAMs in shaping the TME and mediating therapeutic resistance across multiple malignancies. This underscores the necessity of investigating key regulatory factors, such as lactate, which profoundly influence macrophage polarization and immune modulation, thereby playing a pivotal role in tumor progression and therapy resistance.

Lactate, once considered a mere byproduct of anaerobic metabolism, has emerged as a key regulator in tumor metabolism and immune modulation, particularly in TAMs. Lactate-driven metabolic reprogramming influences macrophage polarization, shaping the TME and impacting tumor progression, immune evasion, and therapeutic resistance (9–11, 120). This has established lactate as a promising therapeutic target in cancer treatment.

TAMs exhibit dynamic polarization along a functional spectrum, shaped by metabolic signals from the TME, ranging from pro-inflammatory (M1-like) to anti-inflammatory (M2-like) phenotypes. Within tumors, elevated lactate levels drive macrophages toward an M2-like phenotype, characterized by enhanced immune suppression and tissue remodeling, while preserving their ability to adapt to environmental changes. This M2-like polarization is associated with cytokine secretion (e.g., TGF-β, IL-10) that fosters immune evasion and promotes tumor growth (121). In contrast, M1-like macrophages are associated with anti-tumor immunity, partly through the secretion of pro-inflammatory cytokines like TNF, while their functional state remains influenced by dynamic environmental signals (122). Lactate modulates this polarization shift by influencing not only metabolic processes but also epigenetic mechanisms, particularly histone lactylation. This lactate-induced epigenetic modification plays a key role in macrophage polarization by activating the expression of genes linked to immune suppression and tissue repair (22). Lactate-induced histone modification enhances the expression of M2-associated genes like Arg1, promoting immune tolerance and tissue regeneration (22). Non-histone lactylation also influences key proteins involved in metabolism, linking metabolic shifts to broader cellular functions (82). Lactylation thus emerges as a crucial mechanism by which lactate regulates macrophage behavior and immune responses within the TME.

Given lactate’s profound influence on macrophage polarization and tumor progression, targeting lactate metabolism offers a promising strategy for cancer therapy. Enzymes like LDHA and HK2, crucial for lactate production, are overexpressed in many cancers. Inhibiting LDHA reduces lactate levels, alleviates TME acidification, and reprograms TAMs toward a pro-inflammatory M1-like phenotype, thereby enhancing anti-tumor immunity (25). Similarly, HK2 inhibition disrupts glycolysis, normalizes the TME, and boosts immune activation (87). These findings underscore LDHA and HK2 as promising therapeutic targets. MCTs, especially MCT1 and MCT4, regulate lactate uptake and export in tumor cells and macrophages (26, 95). MCT inhibition blocks lactate transport, inducing metabolic stress in tumor cells and enhancing immune responses. Preclinical studies have demonstrated the potential of MCT inhibitors, such as AZD3965 (MCT1-specific) and syrosingopine (dual MCT1/MCT4), to reduce tumor growth and improve immunotherapy efficacy (44, 95, 96). However, the heterogeneity of tumors and compensatory metabolic pathways pose challenges, highlighting the need for combination therapies.

Beyond macrophage polarization, lactylation has been linked to cancer therapy resistance, particularly in DNA repair. Proteins like MRE11 and XRCC1, involved in homologous recombination, undergo lactylation, which enhances DNA repair and confers resistance to chemotherapy (108, 109). Inhibiting lactylation-regulating enzymes, such as CBP or LDHA, sensitizes tumors to cisplatin and PARP inhibitors, presenting lactylation as a potential target for overcoming therapy resistance (108, 109). This has been particularly demonstrated in glioblastoma, where lactylation-related pathways reverse chemoradiotherapy resistance (109). Notably, lactate modulation has the potential to improve the effectiveness of immunotherapies. Tumor-derived lactate drives TAMs toward an immunosuppressive M2 phenotype, which contributes to resistance against ICIs and CAR-T cell therapies (28, 114–116). By targeting lactate production or lactylation, it is possible to reprogram TAMs to an M1 phenotype, creating a more immunostimulatory environment. Combining lactate-modulating agents with ICIs or CAR-T cell therapies has shown promise in preclinical studies, amplifying anti-tumor immunity and overcoming resistance (28). Despite the therapeutic potential of lactate targeting, several challenges remain, including tumor heterogeneity, metabolic plasticity, and compensatory mechanisms. Moreover, while lactate and lactylation offer novel targets, further research is needed to elucidate the molecular mechanisms underlying their effects on immune modulation and therapy resistance. Identifying biomarkers to predict responses to lactate-targeting therapies and optimizing combination strategies will be essential for enhancing therapeutic efficacy.

In conclusion, lactate-mediated metabolic reprogramming of TAMs is a critical mechanism that drives tumor progression, immune evasion, and therapeutic resistance. By influencing macrophage polarization and modulating immune responses through lactate and lactylation, tumors create an environment conducive to their growth and survival. Targeting lactate metabolism and lactylation offers a promising therapeutic strategy to reprogram the immune landscape, overcome therapy resistance, and enhance the efficacy of cancer treatments. Continued research into the molecular mechanisms of lactate and lactylation will be crucial for developing novel cancer therapies aimed at reshaping the TME and improving treatment outcomes.
